# Investigation of DNA-damage and Chromosomal Aberrations in Blood Cells under the Influence of New Silver-based Antiviral Complex

**DOI:** 10.15171/apb.2016.011

**Published:** 2016-03-17

**Authors:** Evgenii Plotnikov, Vladimir Silnikov, Andrew Gapeyev, Vladimir Plotnikov

**Affiliations:** ^1^ Tomsk Polytechnic University, Tomsk, Russia.; ^2^ Institute of Chemical Biology and Fundamental Medicine, Novosibirsk, Russia.; ^3^ Institute of Cell Biophysics of Russian Acad. Sci., Pushchino, Russia.; ^4^ Polytech Ltd, Tomsk, Russia.

**Keywords:** Genotoxicity, Antiviral compound, Chromosomal aberrations, Silver complex, Comet assay

## Abstract

***Purpose:*** The problem of infectious diseases and drug resistance is becoming increasingly important worldwide. Silver is extensively used as an anti-infective agent, but it has significant toxic side effects. In this regard, it is topical to develop new silver compounds with high biological activity and low toxicity. This work is aimed to study DNA damage and chromosomal aberrations in blood cells under the influence of new silver-based compound of general formula C_6_H_19_Ag_2_N_4_LiO_6_S_2_, with antiviral activity.

***Methods:*** The comet assay was applied for the genotoxic affects assessment on mice blood leukocytes. DNA damage was determined bases on the percentage of DNA in a comet tail (tail DNA), under the influence of silver complex in different concentrations. Genotoxic effect of the tested substance on the somatic cells was determined by chromosomal aberration test of bone marrow cells of mice.

***Results:*** In the course of the experiments, no essential changes in the level of DNA damage in the cells were found, even at highest concentrations. The administration of the substance in doses up to 2.5 g/kg in mice did not cause any increase in the frequency of chromosomal aberration in bone marrow cells.

***Conclusion:*** Taking into account known silver drug genotoxic properties, the use of a given complexed silver compound has possible great advantages for potential applications in the treatment of infectious diseases.

## Introduction

Silver has been widely used as anti-infective agent in various forms and applications. However, silver has significant toxic side effects,^1^ on the other hand, the problem of infectious diseases and drug resistance have become gradually more important. In this regard, it is crucial to develop new silver compounds with high biological activity and low toxicity, especially due to the wide spread of antibiotic-resistant strains of microorganisms. The purpose of this work is to investigate the genotoxic properties of the new silver-based compound with general formula C_6_H_19_Ag_2_N_4_LiO_6_S_2_ by different cytogenetic methods.

Previously it was shown that the compounds of this chemical group have low toxicity and antiviral properties.^2,3^ One of possible mechanisms of heavy metal, including silver, cytotoxic action is that ions enters the cell causing DNA strand breaks.^4^ Silver nanoparticles can also lead to DNA damage causing production of reactive oxygen species and interruption of ATP synthesis.^5^ Thus, the evaluation of genotoxic effects of new silver complex is a necessary parameter for drug development. Currently, the chromosomal aberration assay and the comet assay are a convenient techniques for assessing the genotoxic properties extensively used in nanotoxicology.^6-8^

## Material and Methods

In all experiments, the tested substance was used as an aqueous solution in a wide concentration range. The comet and chromosomal aberration assays were applied for the assessment of genotoxic effects of the substance.

### Chromosomal aberrations assay.

The study of the genotoxic effect of the tested substance was performed on 30 male mice C57BL/6, age 2 - 2.5 months, weight - 20-22g. Before and during the experiments, all groups of animals were kept under natural light conditions with free access to food and water.

The calculation of chromosomal aberrations in bone marrow cells of mice was performed as following.^9^ Tested substance was administered intragastrically one time. The dose range was 1.0 g/kg and 2.5 g/kg. A known cytostatic cyclophosphamide was administered one time, intraperitoneally in the dose of 20 mg/kg, as a positive control. Water for injection was administered intragastrically in an amount of 0.2 ml/20 g body weight of animal as a negative control. The exposure time was 24 hours. For accumulation of metaphase cells, animals were injected intraperitoneally with 0.04% solution of colchicine in dose 0.2 ml/20 g of body weight. Bone marrow was obtained from femoral bone and the samples of metaphase chromosomes were prepared. One hundred cells in metaphase were collected for analysis per each animal. Chromosomal aberrations, including fragments and exchanges, were analyzed in each metaphase cell. Statistical processing was performed using the Student t-test in Fisher transformation.

### Genotoxicity assessment by comet assay. 

Leukocytes fraction was obtained from adult male BALB/c mice (2 months of age, 22–25g in body weight). The mice used in all experiments were maintained under standard condition with free access to food and water. Blood samples stored with 1 mM EDTA as anti-coagulating agent. The blood was diluted 1:7, achieving concentration of leukocytes of 1×10^6^ cells/ml. Silver-containing substance (in water solution) was incubated in aliquots of diluted blood at 37°C for 30 min with stirring. Distilled water in equivalent volume 20% of the sample and hydrogen peroxide at the concentration of 2 µM in blood sample served as a negative control and positive control respectively. Analysis of the level of DNA damage in cells performed using an alkaline comet assay with modification, as described.^7,10^ The method based on analysis of cells with stained DNA.^11^

## Results

**Figure 1:**
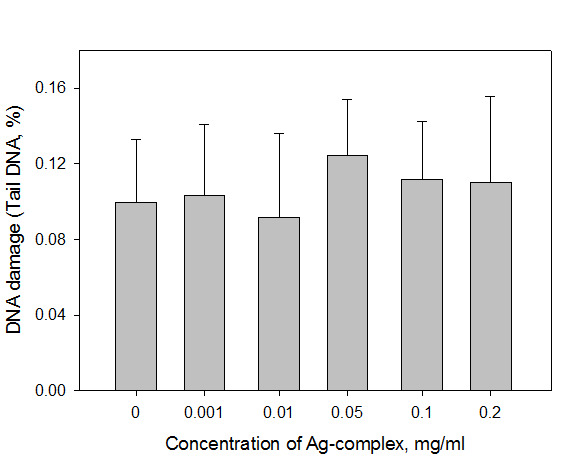
The level of DNA damage in leucocytes under the influence of the silver complex. The incubation of cells in the presence of investigated substance for 30 minutes at 37°C (P<0.05).

**Figure 2:**
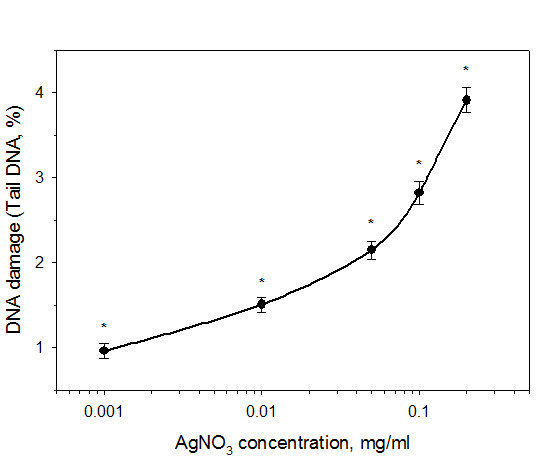
The level of DNA damage in leucocytes under the influence of silver nitrate. The incubation of the cells in the presence of AgNO_3_ for 30 minutes at 37 °C. P<0.05.

The obtained results revealed the absence of genotoxic influence of new silver-based complex in all tested concentrations. Even the highest concentration of 0.2 mg/ml revealed the same level of DNA damage as negative control (Figure 1). Comparative test with other silver salt exhibited high genotoxic action of silver nitrate (Figure 2). Significant differences with negative control were found at concentration range of 0.001 mg/ml and above. The level of DNA damage under the influence of 0.001 mg/ml silver nitrate was about 0.8%, which corresponds to the level of DNA damage by a known oxidative stress agent hydrogen peroxide in a concentration of 2 µM (0.76±0.18%). The comparison based on the content of Ag^+^ ions in the tested substances showed 1000 fold more toxic effect of silver ions in AgNO_3_ (containing 63% silver in molecule) compared to the complex C_6_H_19_Ag_2_N_4_LiO_6_S_2_, (containing 40% silver in molecule), in concentrations, recalculated in silver ions.

As shown in Figure 1, all concentrations in the range of 0.001-0.2 mg/ml caused the same level of DNA damage as in the negative control. The silver-based complex notably reduces toxic effect of silver ions on DNA. Opposite toxic effect is shown in Figure 2 for silver nitrate. The World Health Organization identified the maximum dose for the silver that is 10 g, which causes no detectable adverse effects on human health (NOAEL - No Observable Adverse Effect Level). Safe dose for oral silver exposure is estimated at 5 micrograms/kg/d. This is the amount that can be safely taken daily over a lifetime (70 years) without any adverse effect. However, even a larger dose of silver does not cause any significant side effects.^12,13^

Assessment results of cytogenetic activity of the novel silver complex by the chromosomal aberration test in bone marrow cells of male C57BL/6 mice are shown in Table 1.

The results in the table show that the percentage of aberrant cells in the experiment does not exceed the negative control level.

The administration of the substance in doses of 1.0 g/kg and 2.5 g/kg in mice caused no significant changes in the frequency of chromosomal aberrations in bone marrow cells, indicating the absence of genotoxic effect of the tested complex on somatic cells. This results correlate to comet assay findings (Figure 1). Meanwhile, the percentage of damaged cells in the group of mice, which were administered cyclophosphamide, exceeds the benchmark over 20 times.

**Table 1:** The percentage of chromosome aberrations in bone marrow cells of mice after 24h exposure to the tested substance.

**Substance and Dosage**	**Single fragments, %**	**Paired fragments, %**	**Exchange, %**	**Multiple aberrations, %**	**Damaged cells, %**
Ag-complex, 1.0 g/kg	1,2	0	0	0	1,2
Ag-complex, 2.5 g/kg	2	0,5	0	0	2,5
Cyclophosphamide,20 mg/kg	16	3	0,3	5,3	24,7
Water for Injection 0.2ml/20g	1,2	0	0	0	1,2

## Discussion

The obtained data shows the absence of genotoxic action of silver-contained complex on mice cell (Figure 1, Table 1). Most previous studies revealed, that silver ions as well as silver nanoparticles can cause different level of DNA damage. Silver and other heavy metal salts could cause DNA damage in mammalian cell culture.^4^ There is very limited information regarding genotoxic effects in humans following oral, inhalation or dermal exposure to silver compounds.^13^ Silver nanoparticles could have penetrated the plant system and may have impaired mitosis causing chromosomal aberrations and micronucleus induction that demonstrate genotoxic action.^14^ Silver nanoparticles are considered as a mediator of ROS-induced genotoxicity.^15^ This mechanism is also proved by the experiment of pretreatment with the antioxidant that decreased level of bulky DNA adducts. The same effects were exposed for silver nitrate. Soluble silver salt caused more significant toxic action compared to nanoparticles of all sizes. The mechanism of silver genotoxicity mostly depends on the oxidative stress, the activation of lysosomal acid phosphatase activity and disruption of actin cytoskeleton, but effects mainly expressed for ionic silver impact.^16^ The results of this study showed high level of direct genotoxic action of silver nitrate (Figure 2). That is why it is an important task to produce soluble silver compounds with low toxicity. As shown in Table 1, tested substance caused notable DNA damage level only at high dose. Mainly, there are no contradictions in mechanism of silver DNA damage, but there are many results, which are still contradictive regarding the intensity level of silver genotoxicity. The potential application of known silver compounds could be narrowed by the fact that it is toxic to cells. According to the obtained results, tested silver complex showed low genotoxic properties that make it more appropriate in possible medical applications.

## Conclusion

Thus, new complex C_6_H_19_Ag_2_N_4_LiO_6_S_2_ did not cause DNA damage and did not increase the frequency of chromosomal aberrations in the bone marrow cells. Revealed low genotoxic influence of silver complex require further investigation and preclinical trials.

## Ethical Issues

Not applicable.

## Conflict of Interest

The authors report no conflicts of interest.
